# Differences in tissue-specific insulin resistance between South Asian and Nordic women with prediabetes after gestational diabetes

**DOI:** 10.1007/s00125-025-06546-9

**Published:** 2025-09-24

**Authors:** A. Anita S. Kvist, Archana Sharma, Elisabeth Qvigstad, Naveed Sattar, Jason M. R. Gill, Christina D. Bjørnvall, Tine-Lise Kalleklev, Pushpanjali Shakya, Gerrit van Hall, Frode A. Norheim, Sindre Lee-Ødegård, Kåre I. Birkeland

**Affiliations:** 1 Institute of Clinical Medicine, University of Oslo, Oslo, Norway; 2 Department of Endocrinology, Morbid Obesity and Preventive Medicine, Oslo University Hospital, Oslo, Norway; 3 Department of Endocrinology, Akershus University Hospital, Lørenskog, Norway; 4 School of Cardiovascular and Metabolic Health, University of Glasgow, BHF Glasgow Cardiovascular Research Centre, Glasgow, UK; 5 Department of Nutrition, Institute of Basic Medical Sciences, University of Oslo, Oslo, Norway; 6 Department of Transplantation Medicine, Oslo University Hospital, Oslo, Norway; 7 Clinical Integrative Fluxomics Core, Clinical Biochemistry, Rigshospitalet & Biomedical Sciences, University of Copenhagen, Copenhagen, Denmark

**Keywords:** Adipose tissue, Euglycaemic clamp, Gestational diabetes, Glucose tracer, Insulin sensitivity, Nordics, Prediabetes, South Asians, Transcriptomics

## Abstract

**Aims/hypothesis:**

The aim of this work was to investigate tissue-specific insulin resistance in South Asian and Nordic women with previous gestational diabetes mellitus (pGDM) and to evaluate potential ethnic differences contributing to type 2 diabetes risk.

**Methods:**

A cross-sectional study using a two-step hyperinsulinaemic–euglycaemic clamp with a glucose tracer was conducted to assess insulin sensitivity in muscle, liver and adipose tissue in 19 South Asian and 16 Nordic women with pGDM and prediabetes (impaired glucose tolerance and/or impaired fasting glucose), along with 16 ethnicity-specific control women. We assessed inflammation and mitochondrial genes by mRNA sequencing of adipose tissue.

**Results:**

Both South Asian and Nordic women with pGDM showed reduced total glucose disposal (mainly due to muscle insulin resistance) and hyperinsulinaemia compared with the control group. Endogenous glucose production (mainly due to hepatic insulin resistance) was elevated in Nordics with pGDM, while South Asians with pGDM showed pronounced adipose tissue insulin resistance (reduced suppression of glycerol during clamp). mRNA sequencing of adipose tissue indicated increased tissue inflammation in South Asian women compared with Nordic women with pGDM. Furthermore, we observed a differential response to hyperinsulinaemia in South Asians vs Nordics related to mitochondrial mRNA, such as thymidine kinase 2 (*TK2*). Correlations between adiposity markers and insulin sensitivity also differed by ethnicity, suggesting that the pathways leading to type 2 diabetes may vary across populations.

**Conclusions/interpretation:**

South Asian and Nordic women with pGDM exhibited differences in insulin resistance profiles, with South Asians showing greater adipose tissue insulin resistance and inflammation.

**Graphical Abstract:**

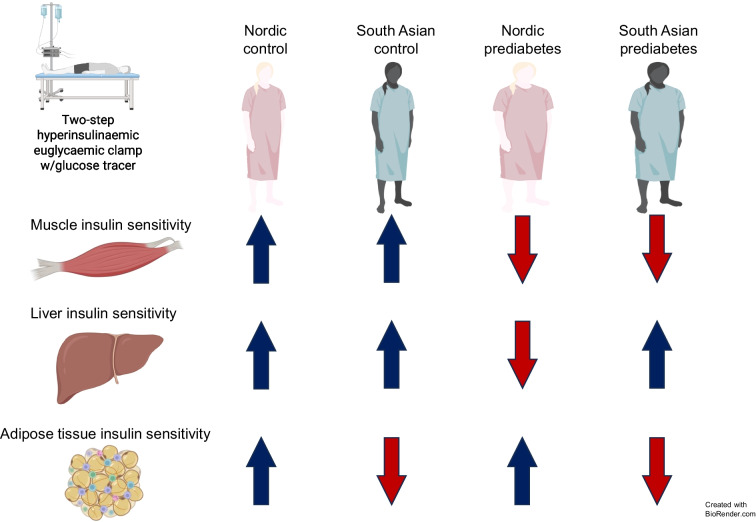

**Supplementary Information:**

The online version of this article (10.1007/s00125-025-06546-9) contains peer-reviewed but unedited supplementary material.


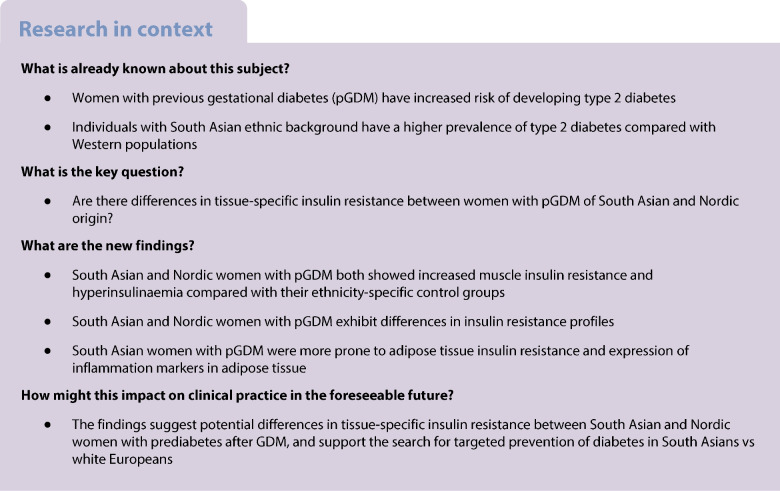


## Introduction

Gestational diabetes mellitus (GDM) is characterised by impaired glucose tolerance during pregnancy. The burden of GDM is not limited to hyperglycaemia during pregnancy; it predisposes women to a series of metabolic disturbances, ultimately contributing to the increased risk of type 2 diabetes [1–6].

Ethnic disparities in the prevalence and progression of type 2 diabetes are well documented, with South Asian individuals having higher rates of adiposity, central obesity and other metabolic disturbances compared with those of European descent [7–11]. Research show that these differences in metabolic risk may be linked to distinct pathways of insulin resistance development. In some previous reports, South Asians present with more visceral fat and lower lean body mass compared with Western Europeans [4, 5, 12, 13], while a recent meta-analysis found that accumulation of liver fat was the dominating finding in South Asians [11, 14].

Insulin resistance, the core feature of type 2 diabetes, is a complex process involving multiple tissues, including muscle, liver and adipose tissue [15, 16]. Muscle insulin resistance is often considered an early pathogenic step, contributing to systemic insulin resistance and subsequent hyperglycaemia [17]. The hyperinsulinaemia associated with muscle insulin resistance can further impact metabolism in the liver and adipose tissue [18]. In the liver, hyperinsulinaemia promotes de novo lipogenesis in the face of excess energy intake, contributing to hepatic insulin resistance [15], while in adipose tissue, it may lead to tissue expansion and inflammation, exacerbating adipose tissue dysfunction [19, 20]. Despite these known mechanisms, the tissue-specific differences in insulin resistance between ethnic groups, particularly in women with previous GDM (pGDM), remain poorly understood.

Previous studies have largely focused on systemic insulin resistance markers [21], with few studies exploring how insulin sensitivity differs across tissues in different ethnic groups using advanced quantification methods [22]. There is a particular gap in understanding how tissue-specific insulin resistance differs between South Asian vs Nordic women following GDM; this may have implications for targeted intervention strategies. Understanding these differences is critical, as they could inform more tailored approaches to reducing type 2 diabetes risk in these high-risk populations.

In this study, we aimed to address this knowledge gap by using state-of-the-art two-step hyperinsulinaemic–euglycaemic clamp with low and high insulin infusion and the use of a glucose tracer to evaluate insulin sensitivity in muscle, liver and adipose tissue in South Asian and Nordic women with or without a history of GDM. Our goal was to determine whether ethnic differences in tissue-specific insulin resistance exist and how these possible differences may reveal specific pathways in the development of type 2 diabetes.

## Methods

### Study design and participants

**Fig. 1:**
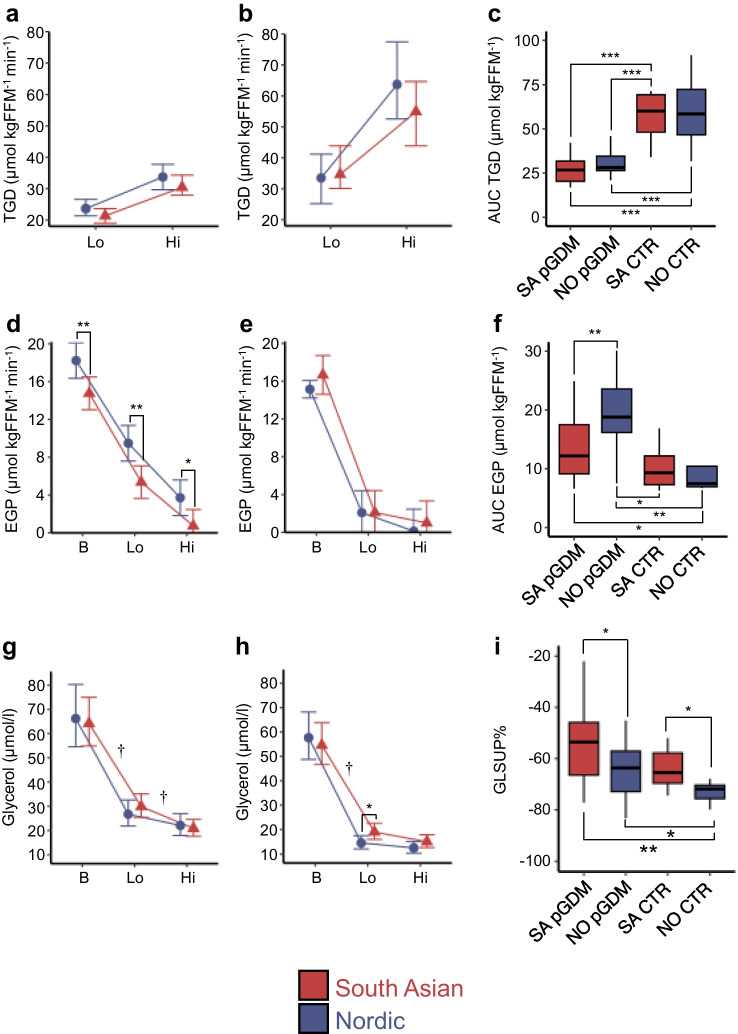
Muscle, liver and adipose tissue insulin sensitivity quantified at low and high rates of insulin infusion during a two-step hyperinsulinaemic–euglycaemic clamp. Red triangles, South Asian women; blue circles, Nordic women. (**a**–**c**) TGD in women with pGDM (**a**) and control women (**b**), and the corresponding AUC for all groups (**c**). (**d**–**f**) EGP in women with pGDM (**d**) and control women (**e**), and the corresponding AUC for all groups (**f**). (**g**–**i**) Glycerol levels in women with pGDM (**g**) and control women (**h**), and GLSUP% for all groups (**i**). Data are presented as mean and 95% CIs or as boxplots with medians, IQR and total range *p<0.05, **p<0.01 and ***p<0.001; ^†^*p*<0.05 group by time interaction effect. B, baseline; CTR, control; FFM, Fat-free mass; Hi, high insulin infusion rate (40 mU m^−2^ min^−1^); Lo, low insulin infusion rate (10 mU m^−2^ min^−1^); NO, Nordic; SA, South Asian

The Diabetes in South Asians (DIASA) 2 study was approved by the South-Eastern Norway Regional Committee for Medical and Health Research Ethics (reference no. 2018/1233). All participants provided written informed consent. A flow chart of the study is presented in electronic supplementary material (ESM) Fig. 1.

Between February 2021 and April 2023 we screened 68 South Asian or Nordic women with pGDM in their last pregnancy, including respective control groups. At inclusion, they all had prediabetes, defined as fasting plasma glucose (FPG) 5.6–6.9 mmol/l or 2 h glucose 7.8–11.0 mmol/l obtained from a standard OGTT with 75 g oral anhydrous glucose in the morning after at least 8 h of fasting. Blood was drawn in the fasting state and 15, 30, 60 and 120 min after the glucose load for glucose and insulin measurements. All but one woman had participated in the DIASA 1 study [7]. In total, 19 South Asian (ten Pakistani, seven Indian, two Sri Lankan) and 16 Nordic (all Norwegian) women fulfilled the inclusion criteria. In addition, eight South Asian (four Sri Lankan, two Pakistani, two Indian) and eight Nordic (six Norwegian, one Swedish, one Icelandic) women with a previous pregnancy without GDM, and FPG <5.6 mmol/l and 2 h glucose ≤7.7 mmol/l were included as control groups. Half of the control women had pregnancies before the index pregnancy. The control women were recruited through advertisements on our hospital’s web page and by spreading the word among participants’ and colleagues’ networks.

In addition to an OGTT, participants underwent a clinical examination including measurements of height, weight, waist and hip circumferences, BP and body composition with bio-impedance (Tanita Body Composition Analyzer BC418; Tokyo, Japan). On a separate day, participants underwent a two-step hyperinsulinaemic–euglycaemic clamp with glucose tracers and adipose tissue samplings.

### Data collection

#### OGTT

We collected blood in either cooled sodium fluoride tubes for glucose analysis (kept on ice until centrifugation at 4°C) within 10 min or serum-separating tubes for analyses of insulin with centrifugation after 30 min.

#### Clinical chemistry

Plasma glucose and lipid levels were measured by enzymatic photometry or colorimetry (Roche Diagnostics, Mannheim, Germany), whole-blood HbA_1c_ by HPLC (G8 HPLC Analyzer; Tosoh Biosciences, Tokyo, Japan) and serum insulin by electrochemiluminescence immunoassay (Cobas e601; Roche Diagnostics). Serum creatinine and estimated GFR (eGFR) was analysed by enzymatic photometry method (Roche Diagnostics) and eGFR was calculated by the CKD-EPI formula. [6,6-^2^H_2_]Glucose and glycerol were measured by LC tandem MS through turbulent flow chromatography (RXT1; Cohesive Technologies, Franklin, MA) combined with tandem MS (Sciex API 3000; Applied Biosystems, Foster City, CA) at the Clinical Metabolomics Core Facility (Rigshospitalet, Copenhagen, Denmark).

#### Two-step hyperinsulinaemic–euglycaemic clamp

Participants fasted, emptied their bladder and their body weight was recorded. Then a venous catheter was inserted into one arm and placed in a heating device for arterialisation of blood. The catheter was kept open with a slow infusion of saline (154 mmol/l NaCl) for blood sampling. Another venous catheter was inserted into the other arm for infusion of insulin and glucose. A bolus dose of 270 mg of [6,6-^2^H_2_]glucose was given intravenously followed by a continuous infusion of labelled glucose at 2.8 mg/min, which was kept unchanged during the clamp. After a stabilisation phase of 120 min, the clamp was started with an infusion of Insulin Actrapid (Novo Nordisk, Bagsvaerd, Denmark) at a rate of 10 mU m^−2^ min^−1^ (‘low insulin’). A variable infusion of glucose 200 mg/ml enriched with 8 mg [6,6-^2^H_2_]glucose was continually adjusted to maintain euglycaemia. After 90 min, the insulin infusion rate was increased to 40 mU m^−2^ min^−1^ (‘high insulin’) for another 90 min. Endogenous glucose production (EGP) was estimated during the last 30 min of the stabilisation phase. During both low and high insulin infusion, the total glucose disposal (TGD) was estimated as the total rate of disappearance, including both the exogenous glucose infusion rate and the EGP estimated by the stable isotope dilution method during the last 30 min of each step [23]. During the clamp, glucose was measured using YSI 2300 STAT Analyzer (Yellow Springs Instruments, Yellow Springs, OH, USA).

#### Adipose tissue sampling

Adipose tissue was harvested before and after the clamp using an aspiration technique of periumbilical fat from only the women with pGDM. Lidocaine (without adrenaline [epinephrine]) was injected before the procedure. Aliquots of snap-frozen adipose tissue were stored at –80°C until RNA isolation, cDNA synthesis and mRNA sequencing.

#### Adipose tissue RNA isolation

Adipose tissue aliquots were homogenised in tubes containing glass beads and QIAzol (QIAGEN, Hilden, Germany) by vigorous shaking (2 × 5000 rev/min at intervals of 30 s) using Precellys 24 (Bertin Technologies, Montigny-le-Bretonneux, France). The lysate was centrifuged at 12,000 *g* for 10 min at 4°C to eliminate lipids. After addition of chloroform, the samples were centrifuged at 12,000 *g* for 15 min at 4°C. The water-soluble phase was loaded onto the NucleoSpin RNA isolation kit for subsequent RNA isolation (Macherey–Nagel, Allentown, PA, USA). RNA purity and concentration were measured on a NanoPhotometer NP80 (Implen, Munich, Germany). RNA integrity was determined using a Bioanalyzer RNA 6000 Nano (Agilent Technologies), according to the manufacturer’s instruction.

#### Adipose tissue mRNA sequencing

Total RNA from adipose tissue was sequenced at the Norwegian Sequencing Centre (Oslo University Hospital, Ullevål, Oslo). Downstream analysis was performed using Salmon v1.10.1 (https://salmon.readthedocs.io/) for alignment and gene-level expression. edgeR v4.4.2 was thereafter used for differential expression analyses in R v4.4.2 (https://www.R-project.org/, R Foundation for Statistical Computing). Pathway analyses were performed using Reactome v1.89.0 in the clusterProfiler R v4.14.6 package. Clustering was done using the pheatmap v1.0.13 R package. mRNA levels are presented as counts per million after trimmed mean of *M* values (TMM) normalisation.

We used a volcano plot to show ethnic differences in mRNA expression at baseline. The logarithmic transformations used in volcano plots are necessary for comprehensive and symmetrical representation of the large amount of data. Red (higher mRNA levels in South Asians) and blue (higher mRNA levels in Nordics) coloured points in the plots indicate statistical significance (*p*<0.05), whereas grey points signify statistical insignificance (*p*>0.05). mRNA pathway analyses were used to explore responses to insulin infusion during clamp. We used heatmaps to illustrate multiple correlations, with positive correlations shown in red and negative correlations shown in blue. The intensity of the colour in the heatmaps reflects the strength of the correlation. We used dendrograms along the *y*-axis of the heatmaps to illustrate the association between mRNAs, with clustered mRNAs represented using a colour-coded scheme.

### Statistics

Participant characteristics were compared between the groups using Wilcoxon tests and data are presented as medians and IQRs. Responses to the clamp were modelled using linear mixed models using the lme4 v1.1.37 R package. Data were rank-transformed to obtain normality, but back-transformed for visualisation of marginal means and 95% CIs. AUC was calculated using the trapezoid rule. Ethnic differences in associations between insulin levels and tissue-specific insulin sensitivity were assessed using linear regression. Spearman’s correlations were used for bivariate relationships.

## Results

The characteristics of the participants are shown in Table 1. The women in the two pGDM groups were older and had a longer time since index pregnancy than the women in their respective control groups. South Asian women were shorter than Nordic women and had lower total and truncal fat-free mass. Women with pGDM had higher BMI, body fat mass, waist-to-height ratio (WHtR), HbA_1c_ and fasting glucose compared with controls, as well as increased waist and hip circumference and lower HDL-cholesterol levels. The South Asian women with pGDM displayed higher HbA_1c_ levels than Nordic women with pGDM.

**Table 1:** Key clinical characteristics for the participating women

Characteristic	pGDM SA	pGDM NO	Control SA	Control NO
*N*	19	16	8	8
Age, years	36.0 (6.5)^§^	37.0 (9.5)^§^	31.0 (3.5)*‡	34.0 (2.8)
Height, cm	156 (4.8)^‡†^	167 (6.1)^§^	159 (5.3)^‡†^	164 (9.3)*^§^
Weight, kg	74.7 (20.4)^§^	78 (13.2)^§†^	61 (15.9)*^‡^	62.7 (7.6)^‡^
Waist, cm	95.5 (16.5)^§†^	88.9 (14.1)^§†^	74.8 (18.6)*^‡^	74.5 (6.3)*^‡^
Hip, cm	105 (13.2)^§†^	111 (14.0)^§†^	92.8 (16.9)*^‡^	90.2 (7.9)*^‡^
BMI, kg/m^2^	30.2 (7.6)^†^	28.4 (4.2)^§†^	24.4 (8.3)*^‡^	22.3 (2.8)*^‡^
WHR	0.90 (0.10)^§†^	0.85 (0.12)	0.82 (0.04)*	0.82 (0.09)*
WHtR	0.61 (0.12)^§†^	0.55 (0.08)^§†^	0.47 (0.12)*^‡^	0.45 (0.02)*^‡^
Body fat, %	40.8 (7.7)^§†^	39.2 (6.2)^§†^	30.8 (13.8)*^‡^	25.2 (5.8)*^‡^
Body fat, kg	28.7 (12.6)^§†^	30.6 (8.7)^§†^	18.6 (13.0)*^‡^	15.7 (4.1)*^‡^
Truncal fat, %	37.2 (7.8)^†^	37.2 (7.17)^§†^	26.3 (16.1)^‡^	21.9 (6.3)*^‡^
Truncal fat, kg	14 (6.7)^§†^	15.8 (5.1)^§†^	8.5 (7.3)*^‡^	7.3 (2.2)*^‡^
FFM, kg	44.6 (8.6)^‡^	49.0 (3.8)^§^	40.4 (4.5)^‡†^	45.1 (5.2)^§^
Truncal FFM, kg	25.3 (4.8)^‡^	27.3 (2.7)^§^	23.0 (2.5)^‡†^	26.0 (3.1)^§^
Time since index pregnancy, months	34 (22)^§†^	33 (25)^§†^	21 (6.8)*^‡^	22 (9.3)*^‡^
Systolic BP, mmHg	112 (8.5)	111 (15.5)	104 (10.6)	107 (18.4)
Diastolic BP, mmHg	72 (10.8)	76 (13.9)	71 (6.4)	65 (17.6)
HR, beats/min	73 (15.5)	71 (16.4)	72 (8.88)	65 (16.8)
HbA_1c_, mmol/mol	41 (3)^§†^	38(3.5)*^†^	36 (3.3)*	34 (3.3)*^‡^
HbA_1c_, %	5.9 (0.3)^§†^	5.6 (0.35)*^†^	5.4 (0.35)*	5.3 (0.3)*^‡^
FPG, mmol/l	5.9 (0.5)^§†^	5.6 (0.5)^§†^	5.1 (0.4)*^‡^	5.1 (0.6)*^‡^
f-Insulin, pmol/l	95.0 (56.5)^§†^	84.0 (68.7)^†^	65.5 (29.8)*^†^	38.5 (19.8)*^§^
GIR, µmol kg^−1^ min^−1^	29.6 (24.3)^§†^	34.0 (10.9)^§†^	57.5 (23.8)*^‡^	64.5 (29.2)*^‡^
Total cholesterol, mmol/l	4.2 (0.5)	4.1 (1.3)	4.1 (0.7)	3.9 (0.5)
HDL-cholesterol, mmol/l	1.0 (0.4)^§†^	1.1 (0.3)^§†^	1.4 (0.2)*^‡^	1.7 (0.4)*^‡^
LDL-cholesterol, mmol/l	2.7 (0.6)	2.8 (0.7)^†^	2.4 (0.6)	2.0 (0.5)^‡^
Triglycerides, mmol/l	1.0 (0.7)^†^	1.0 (0.9)^†^	0.7 (0.7)	0.6 (0.1)*^‡^
Creatinine, µmol/l	54.5 (9.5)^†^	59.0 (6.3)	57.5 (14.5)	65.0 (7.3)*
eGFR, ml/min per 1.73 m^2^	116 (7.3)	110 (13.5)	118 (16)	107 (15.8)

Data are presented as median (IQR)

^*^p<0.05 vs pGDM SA; ^‡^*p*<0.05 vs pGDM NO; ^§^*p*<0.05 vs Control SA; ^†^*p*<0.05 vs Control NO (using Wilcoxon tests)

FFM, fat-free mass; f-Insulin, fasting serum insulin; GIR, glucose infusion rate; NO, Nordic; SA, South Asian

### Tissue-specific insulin sensitivity

TGD, mainly reflecting muscle insulin sensitivity, increased from the low to the high insulin infusion rates (Fig. 1a, b), and were significantly lower in women with pGDM than in controls (Fig. 1c), as expected. No ethnic differences were observed in insulin sensitivity. Fasting EGP, mainly reflecting liver insulin sensitivity, was significantly elevated in Nordic women with pGDM compared with their control counterparts and compared with South Asian women with pGDM; fasting EGP did not differ significantly between the two groups of South Asian women (Fig. 1d–f). EGP fell in response to insulin infusion in all groups as expected (Fig. 1d, e). The reduction in plasma glycerol levels, mainly reflecting adipose tissue insulin sensitivity, was significantly less in South Asian than in Nordic women, suggesting lower adipose tissue insulin sensitivity in South Asian women (Fig. 1g–i). Serum insulin levels were higher both in the fasting state and during low and high insulin infusion in the South Asian control group of women compared with the Nordic control group (Table 2). Adjusting for group differences in insulin levels during the clamp did not alter the results.

**Table 2:** Insulin levels and tissue-specific insulin sensitivity adjusted for insulin levels

Variable	pGDM			Control		
	SA	NO	*p* value^a^	SA	NO	*p* value^b^
S-insulin (basal^c^), pmol/l	105.8 (85.0, 129.0)	95.0 (75.0, 110.3)	0.531	56.0 (46.0, 76.0)	32.0 (26.0, 51.0)	0.021
S-insulin (low infusion rate^d^), pmol/l	198.3 (186.3, 235.3)	187.0 (165.0, 205.0)	0.295	218.0 (166.7, 335.0)	143.0 (128.0, 165.0)	0.002
S-insulin (high infusion rate^e^), pmol/l	590.5 (576.3, 686.0)	542.7 (509.3, 589.0)	0.054	509.7 (460.0, 637.7)	436.7 (412.0, 476.7)	0.013
TGD/I^f^, μmol (kg FFM)^−1^ min^−1^	73.2 (48.1, 98.4)	56.8 (29.3, 84.2)	0.375	95.8 (73.2, 118.5)	92.7 (70.1, 115.4)	0.840
EGP/I^g^, μmol (kg FFM)^−1^ min^−1^	13.2 (11.9, 14.6)	16.9 (15.4, 18.4)	0.001	15.7 (12.4, 19.1)	12.7 (9.3, 16.1)	0.192
GLSUP%/I^h^, %	−56.7 (−70.5, −42.9)	−77.6 (−92.6, −62.6)	0.045	−29.8 (−39.9, −19.8)	−38.7 (−48.8, −28.6)	0.204

Data are presented as mean (95% CI)

The insulin sensitivity values represent the estimated effect on the original scale after adjusting for insulin. This was achieved by including insulin as a covariate in the regression model, ensuring that the reported values account for its influence while preserving interpretability

^a^*p* value is given for pGDM SA vs pGDM NO

^b^*p* value is given for CTR SA vs CTR NO

^c^Basal insulin is fasting serum insulin levels

^d^Low infusion rate is the mean of three measurements during steady-state with 10 mU m^−2^ min^−1^ insulin infusion

^e^High infusion rate is the mean of three measurements during steady-state with 40 mU m^−2^ min^−1^ insulin infusion

^f^TGD/I represents TGP at 40 mU m^−2^ min^−1^ adjusted for insulin levels during high infusion rate

^g^EGP/I represents fasting EGP adjusted for basal insulin

^h^GLSUP%/I % represents reduction in glycerol levels from basal to low infusion rate during clamp adjusted for insulin levels at the low infusion rate step

FFM, fat-free mass; NO, Nordic; SA, South Asian S-insulin, serum insulin

In sensitivity analyses, TGD remained significantly different when comparing women with pGDM and their control counterparts after adjustment for age, BMI and HbA_1c_, although the effect size was attenuated (ESM Table 1). The results for EGP also remained largely unchanged after adjustment for age, BMI and HbA_1c_ but the difference between South Asians and Nordics with pGDM increased (ESM Table 2). For glycerol suppression, the difference between Nordic women with pGDM vs the control women was fully explained by BMI (ESM Table 3). We note that when adjusted for BMI, South Asian control women displayed significantly lower glycerol suppression than Nordics with pGDM (ESM Table 3).

### Association between adiposity and tissue-specific insulin sensitivity

**Fig. 2:**
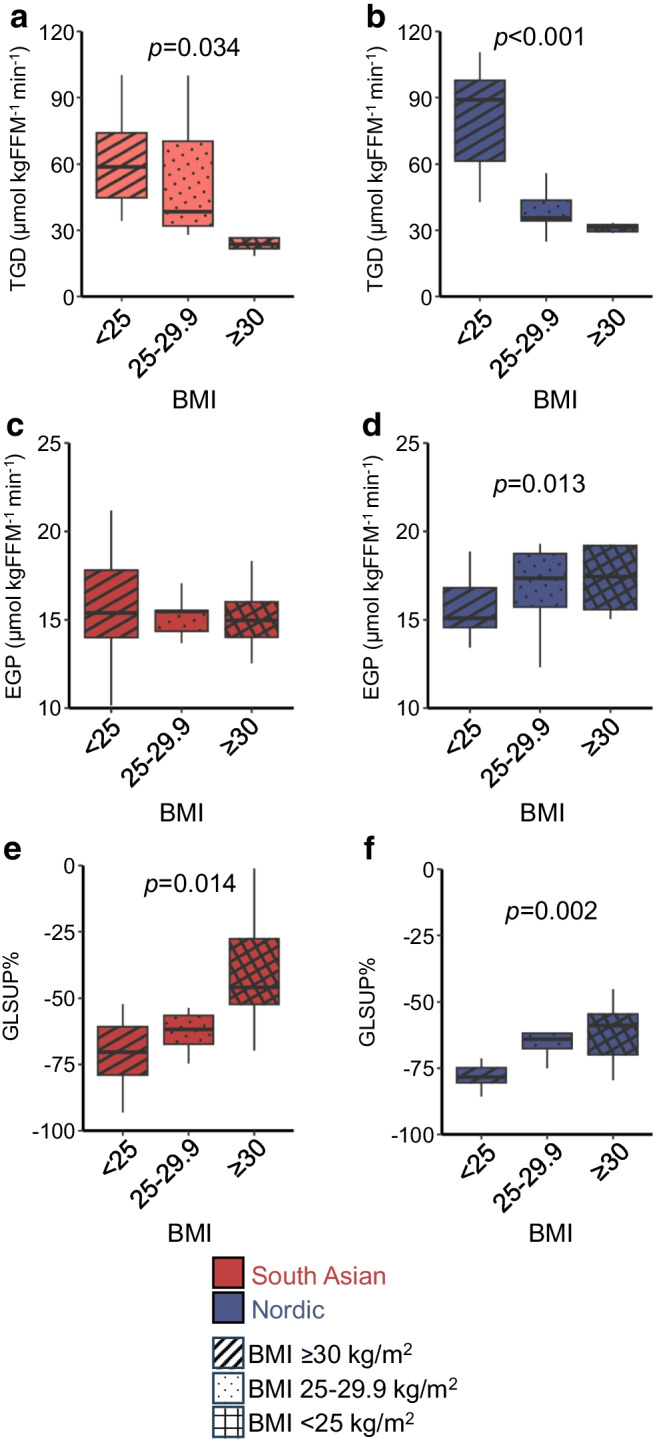
Adiposity and tissue insulin sensitivity. Associations between tissue-specific insulin sensitivity and BMI categories in South Asian women (*n*=8, BMI<25 kg/m^2^; *n*=9, BMI 25–29.9 kg/m^2^; *n*=10, BMI≥30 kg/m^2^) and Nordic women (*n*=9, BMI<25 kg/m^2^; *n*=9, BMI 25–29.9 kg/m^2^; *n*=6, BMI≥30 kg/m^2^) across pGDM and control groups. (**a**, **b**) TGD in South Asians (**a**) and Nordics (**b**). (**c**, **d**) EGP in South Asians (**c**) and Nordics (**d**). (**e**, **f**) Suppression of glycerol levels during clamp in South Asians (**e**) and Nordics (**f**). Box plots show medians, IQRs and total range. *p* values are for one-way ANOVA across BMI groups. FFM, fat-free mass

To explore the relationship between adiposity and tissue-specific insulin sensitivity in the two ethnic groups, we examined the data by BMI categories and included both women with pGDM and control women (Fig. 2).

We observed a clear negative relationship between BMI and TGD (Fig. 2a, b) and a clear positive relationship between BMI and lower suppression of glycerol levels (Fig. 2e, f) in both South Asian and Nordic women. However, a positive relationship between BMI and EGP was only seen in Nordics, and not in South Asians (Fig. 2c, d). Substituting BMI with WHtR or truncal fat mass (markers of visceral fat mass) did not change the results (data not shown).

We supplied the analysis in Fig. 2 by using a regression approach using TGD, EGP or per cent suppression of glycerol levels during clamp (GLSUP%) as dependent variables, and BMI by ethnicity, adjusted for pGDM, as predictors (ESM Table 4). Briefly, BMI was a significant predictor of TGD and GLSUP%, but not for EGP. However, BMI by ethnicity was a significant predictor of EGP. In summary, these results are similar to the stratified approach shown in Fig. 2.

### Metabolic markers and tissue-specific insulin sensitivity

**Fig. 3:**
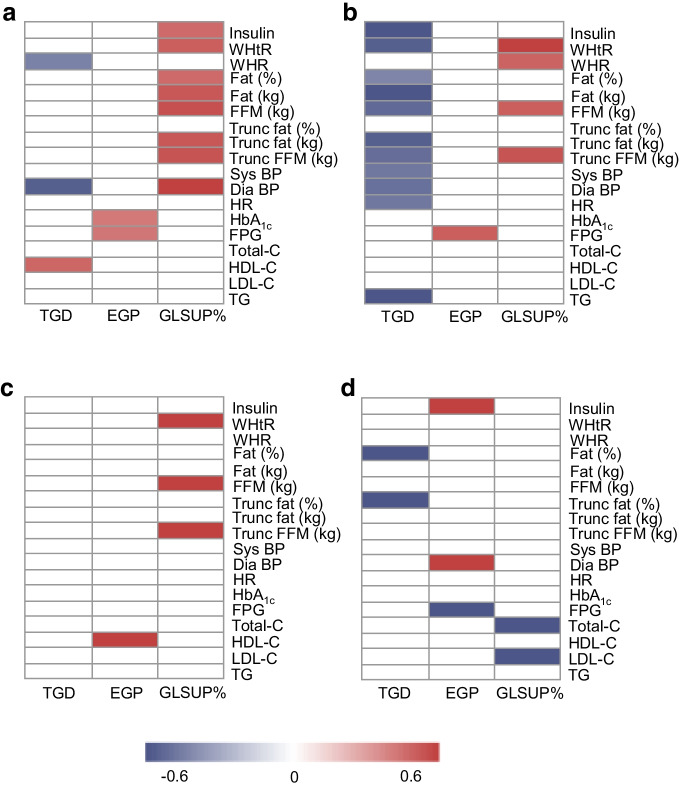
Spearman’s correlations between metabolic markers and tissue-specific insulin sensitivity in South Asian women with pGDM (**a**), Nordic women with pGDM (**b**), South Asian control women (**c**) and Nordic control women (**d**). Heatmaps show correlations as grades of red (positive) and blue (negative). Correlations with* p*>0.05 are shown as white. ALAT, alanine aminotransferase; ASAT, aspartate aminotransferase; Dia BP, diastolic BP; FFM, fat-free mass; HDL-C, HDL-cholesterol; HR, heart rate; LDL-C, LDL-cholesterol; Sys BP, systolic BP; TG, triglycerides; Total-C, total cholesterol; Trunc, truncal

Correlation analysis of metabolic markers revealed potential differences in the relationships between tissue-specific indices of insulin sensitivity in South Asian vs Nordic women (Fig. 3). In women with pGDM, South Asians displayed strong positive relationships between adipose insulin resistance and fat mass, whereas these associations were not seen in Nordics (Fig. 3a, b). On the other hand, Nordics displayed strong negative relationships between muscle insulin resistance (TGD) and fat mass (Fig. 3a, b). EGP was correlated with FPG in both groups. The associations between metabolic markers and insulin sensitivity were weaker in control women than in women with pGDM (Fig. 3c, d). Details are provided in ESM Tables 5–10. Although many correlations were numerically different in South Asian vs Nordic women with pGDM, most of them did not reach statistical significance in formal interaction analysis, probably due to low statistical power (ESM Tables 11–12).

### Adipose tissue inflammation and insulin sensitivity

**Fig. 4:**
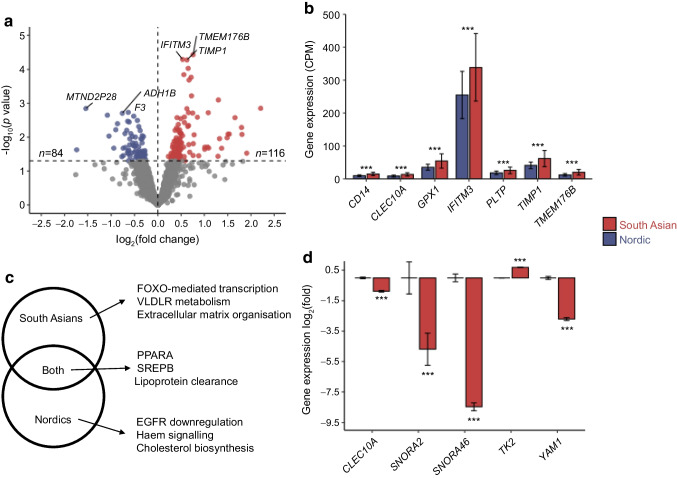
Adipose tissue mRNA sequencing in South Asian vs Nordic women with pGDM at baseline and in response to insulin. (**a**) Volcano plot showing ethnic differences in mRNA expression at baseline. On the *x*-axis, data points >0 and <0 indicate association with South Asian and Nordic ancestry, respectively. On the *y*-axis, large values represent low *p* values. The logarithmic transformations used in volcano plots are necessary for comprehensive and symmetrical representation of the large amount of data. Red (South Asian women) and blue (Nordic women) data points indicate statistical significance (*p*<0.05); grey points signify statistically insignificant associations (*p*>0.05). (**b**) mRNAs with the highest statistical differences between ethnic groups at baseline. (**c**) Pathway analyses showing the most important pathway responding to insulin infusion in the two ethnic groups. (**d**) Differential responses in mRNA expression in response to insulin infusion between the ethnic groups. ***p<0.001. Bar plots present mean and 95% CIs. CPM, counts per million; PPARA, peroxisome proliferator-activated receptor α; SREBP, sterol regulatory element-binding protein; VLDLR, VLDL receptor; EGFR, Epidermal Growth Factor Receptor Pathway

In women with pGDM, we observed differences in mRNA expression between South Asian and Nordic women at baseline (Fig. 4a). The result was similar when adjusted for BMI but the number of mRNAs differing between South Asians and Nordics increased from 200 to 318. These results included increased expression of macrophage and inflammation markers in South Asians (Fig. 4b).

Insulin infusion altered the gene expression in expected pathways, such as peroxisome proliferator-activated receptor α (PPARA), sterol regulatory element-binding protein (SREPB) (white adipocyte differentiation) and lipid metabolism pathways, in both groups (Fig. 4c). However, different responses were also seen within each ethnic group (Fig. 4c). For example, South Asians displayed different responses in markers of tissue remodelling and VLDL metabolism as compared with Nordics (Fig. 4c). Some of the mRNAs with different responses to insulin infusion included those related to mitochondria (thymidine kinase 2; *TK2*, Fig. 4d) and macrophage markers (c-type lectin domain containing 10A; *CLEC10A*, Fig. 4d).

**Fig. 5:**
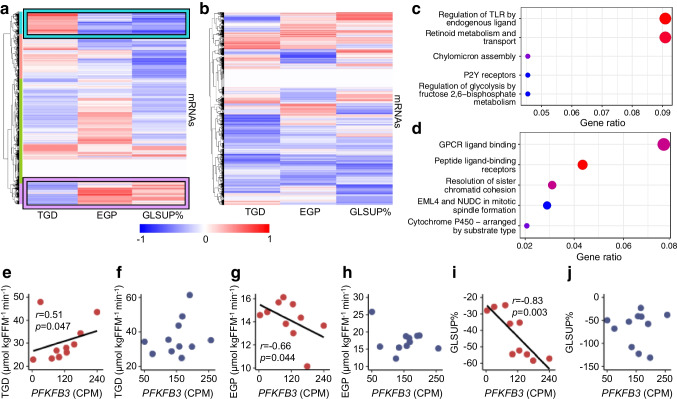
Adipose tissue mRNA sequencing vs tissue-specific insulin sensitivity in South Asian vs Nordic women with pGDM. (**a**, **b**) Heatmaps of clustered Spearman’s correlations between mRNA levels at baseline and insulin sensitivity measured during clamp in South Asian (**a**) and Nordic (**b**) women. Positive correlations are shown in red; negative correlations are shown in blue. The intensity of the colour reflects the strength of the correlation, as indicated by the colour key. The dendrograms illustrate the relationships between mRNAs, with clustered mRNAs represented using a colour-coded scheme. The cyan and purple clusters are highlighted with boxes. (**c**, **d**) Pathways associated with the cyan (**c**) and purple clusters (**d**). Bubble size represents the number of overlapping genes, and the colour scale (red=more significant, blue=less significant) indicates the significance level. (**e**–**j**) Correlations between mRNA levels of the key regulator *PFKFB3* (involved in fructose 2,6-bisphosphate metabolism) and TGD in South Asians (**e**) and Nordics (**f**), EGP in South Asians (**g**) and Nordics (**h**), and GLSUP% in South Asians (**i**) and Nordics (**j**). CPM, counts per million; EML4, echinoderm microtubule-associated protein-like 4; FFM, fat-free mass; GPCR, G-protein coupled receptor binding; NUDC, nuclear migration protein C; P2Y, purinergic 2Y; TLR, toll-like receptor

Furthermore, large-scale clustering analyses of adipose tissue mRNA levels and insulin sensitivity revealed distinct South Asian-specific gene clusters (Fig. 5a, b), e.g. one gene cluster (cyan colour in Fig. 5a) was positively correlated to TGD and negatively correlated to EGP and GLSUP% (Fig. 5a). This gene cluster was not detected in Nordics (Fig. 5b). The gene cluster was associated with Toll-like receptor signalling and glycolysis (Fig. 5c). Another gene cluster (purple colour in Fig. 5a) was negatively correlated to TGD and positively correlated to EGP and GLSUP% (Fig. 5a), was related to G-protein coupled receptor binding (Fig. 5d) and was involved in several regulatory long non-coding RNAs (lncRNA) (Fig. 5d). Similarly, this gene cluster was not found in Nordics (Fig. 5b). A follow-up analysis of the key regulator 6-phosphofructo-2-kinase/fructose-2,6-bisphosphatase 3 (encoded by *PFKFB3*) in glycolysis revealed strong correlations in South Asians and no correlations in Nordics (Fig. 5e–j).

## Discussion

State-of-the-art quantification of tissue-specific insulin sensitivity, using a two-step hyperinsulinaemic–euglycaemic clamp with a glucose tracer, revealed potentially differing patterns of insulin resistance in South Asian and Nordic women with pGDM. The observation of increased muscle insulin resistance in women with pGDM aligns with prior studies indicating that lean first-degree relatives of individuals with type 2 diabetes often show muscle insulin resistance decades before diabetes onset [24], making it an early marker of systemic insulin resistance and a precursor to hyperglycaemia and type 2 diabetes [17]. The approximately twofold reduction in TGD, mainly reflecting muscle insulin resistance, observed in women with pGDM compared with control women is therefore likely a key factor in their elevated diabetes risk.

Our findings also show that hyperinsulinaemia accompanied muscle insulin resistance in women with pGDM, probably as a compensatory mechanism [15, 16]. However, hyperinsulinaemia may also contribute to increased hepatic insulin resistance via enhanced de novo lipogenesis, as excess nutrients are directed to the liver when muscle nutrient uptake is compromised [18, 25]. This mechanism aligns with our observation of elevated EGP in Nordic women with pGDM, a measure of hepatic insulin resistance [26]. We add novel data to this field by demonstrating that EGP remained elevated even after adjusting for insulin levels, further suggesting hepatic insulin resistance as an important early risk factor of type 2 diabetes in Nordic women with pGDM. We are not aware of previous literature with similar findings.

Hyperinsulinaemia may also drive adipose tissue expansion and inflammation [19], potentially leading to adipose tissue insulin resistance characterised by increased lipolysis and release of glycerol and NEFA [12]. In a recent review, the authors suggested that there may not be increased visceral fat in South Asians compared with white Europeans [11, 14]. Even if this is correct, this is still compatible with our findings of more pronounced insulin resistance in adipose tissue. Elevated lipolysis may promote ectopic lipid deposition in muscle and liver, worsening insulin resistance in these tissues and increasing diabetes risk [15, 16]. A previous study reported that South Asians seem to store more ectopic fat in liver compared with white Europeans with similar BMI levels [11], and we previously reported elevated non-alcoholic fatty liver disease liver fat score (NAFLD-LFS) in South Asian women, which contributed to the ethnic differences in glucose metabolism [12].

Notably, elevated EGP (primarily reflecting hepatic insulin resistance) was more pronounced in Nordic women, whereas reduced GLSUP% (indicating adipose tissue insulin resistance) was potentially more characteristic of South Asians. These findings highlight the complex and heterogeneous nature of insulin resistance and its association with type 2 diabetes risk across different ethnicities. In Nordic women, muscle insulin resistance and hyperinsulinaemia may drive hepatic insulin resistance, whereas in South Asian women, hyperinsulinaemia may contribute to their hypertrophic adipocytes and adipose tissue dysfunction [20, 27].

This raises the question: what factors drive these ethnic differences in tissue-specific insulin resistance? Thus, we correlated metabolic markers to the measures of insulin sensitivity. This led us to the finding that the accumulation of fat was most strongly correlated with TGD (muscle insulin resistance) in Nordics, while it had the strongest correlation to GLSUP% (adipose tissue insulin resistance) in South Asians. Hence, these differences may imply different ethnicity-specific pathogenic pathways toward type 2 diabetes, potentially influenced by lifestyle, genetic factors or uncharacterised metabolic mediators. These findings need to be validated in further studies.

In line with previous results, adipose tissue insulin resistance was more pronounced in South Asians than in Nordics [12]. Therefore, we performed mRNA sequencing to further characterise ethnic differences in adipose tissue in women with pGDM. The results indicated that immune-cell-related mRNA levels in adipose tissue [28, 29] were increased among South Asians. In addition, the response to insulin infusion differed between the ethnicities in gene markers such as those for mitochondrial function (*TK2*) [30] and inflammation (forkhead box O1 [FOXO1]-related pathways and *CLEC10A*) [31]. Clustering analysis of mRNAs and insulin sensitivity markers revealed distinct patterns in South Asians but not in Nordics. For example, we identified an insulin sensitivity-related gene cluster associated with Toll-like receptor signalling and glycolysis in South Asians, likely reflecting the presence of more immune cells in adipose tissue [32]. The observation that FOXO-mediated pathways were important in the response of South Asians to insulin infusion, which generally promotes macrophage M1 polarisation, might indicate the presence of proinflammatory macrophages in adipose tissue in South Asians [28, 29]. This is in line with the differential correlation results for *PFKFB3* and insulin sensitivity between the two ethnic groups, since 6-phosphofructo-2-kinase/fructose-2,6-bisphosphatase 3 is a glycolysis-related mediator linked to M1 polarisation [33]. Furthermore, our data also implied differences in adipose tissue mRNA levels related to extracellular matrix remodelling, which may be linked to fibrosis and systemic insulin resistance in South Asians [34].

Hence, our findings may point to a possible predisposition in South Asians for adipose tissue insulin resistance that might be a characteristic feature [12]. Notably, South Asian women are known to have near double the c-reactive protein levels of Europeans for reasons that are not well understood, although this may be linked to greater adipose tissue inflammatory activity [35].

Nevertheless, as a cross-sectional study, our findings provide only a snapshot of insulin resistance across tissues, limiting our ability to define the temporal sequence of insulin resistance onset. It is possible that insulin resistance arises simultaneously across various tissues, with complex, bidirectional influences. For instance, adipose tissue insulin resistance in South Asians may exacerbate muscle insulin resistance and eventually impair hepatic function via elevated hepatic lipid accumulation. Conversely, increased liver fat in Nordics may drive hepatic insulin resistance, subsequently affecting muscle and adipose tissue. Thus, our findings may reflect an ethnic divergence in the pattern of insulin resistance, though it remains uncertain whether these patterns develop sequentially or in parallel, or whether they partially differ due to Nordic women having to gain more fat to develop diabetogenic status in or out of pregnancy compared with South Asian women. Longitudinal studies are needed to clarify these potential pathways and interactions over time. Although EGP mainly comes from the liver, the kidneys also play a role. Further limitations to our study are the lack of data on diet and physical activity and a number of differences between the pGDM and control women that may associate with our main outcomes, including a variable number of previous pregnancies before the index pregnancy. We also performed a large number of statistical tests; however, rather than adjusting for multiple testing (e.g. using Bonferroni correction), we emphasised biological plausibility and consistency across related outcomes to aid interpretation. We acknowledge that our results should be interpreted with care and validated in independent cohorts. Breastfeeding may impact insulin sensitivity in women for a long time after stopping [36] but we did not have the opportunity to test this in our study.

In conclusion, our findings indicate that South Asian and Nordic women with a history of GDM seem to exhibit different insulin resistance profiles, which may potentially contribute to differing risks of developing type 2 diabetes. These differences underscore the need for further research into genetic predisposition and tissue-specific mechanisms to inform tailored prevention strategies for high-risk populations.

## Supplementary Information

Below is the link to the electronic supplementary material.

Supplementary file1 (PDF 502 KB)

## Data Availability

The datasets generated during and/or analysed during the current study are available from the corresponding author on reasonable request.

## Abbreviations

DIASA: Diabetes in South Asians
eGFR: Estimated GFR
EGP: Endogenous glucose production
FOXO1: Forkhead box O1
FPG: Fasting plasma glucose
GDM: Gestational diabetes mellitus
GLSUP%: Suppression of glycerol levels during clamp
pGDM: Previous GDM
TGD: Total glucose disposal
WhtR: Waist-to-height ratio

## Authors’ relationships and activities

NS has received consulting fees from AbbVie, Amgen, AstraZeneca, Boehringer Ingelheim, Carmot Therapeutics, Eli Lilly, GlaxoSmithKline, Hanmi Pharmaceuticals, Menarini-Ricerche, Metsera, Novartis, Novo Nordisk, Pfizer, and Roche; speaker honoraria from Abbott Laboratories, AbbVie, Amgen, AstraZeneca, Boehringer Ingelheim, Eli Lilly, Novo Nordisk, and Roche; and grant support paid to his university from AstraZeneca, Boehringer Ingelheim, Novartis, and Roche outside the submitted work. All other authors declare that there are no relationships or activities that might bias, or be perceived to bias, their work.
