# Supplementary material for: Differences in tissue-specific insulin resistance between South Asian and Nordic women with prediabetes after gestational diabetes
Source: Diabetologia. 2025 Sep 24;68(12):2696–708. doi: 10.1007/s00125-025-06546-9 (PMC12594657; doi:10.1007/s00125-025-06546-9)
Supplement: Supplementary file 1 — Supplementary file1 (PDF 502 KB) [file 125_2025_6546_MOESM1_ESM.pdf]

ESM Table 1.

Sensitivity analyses for TGD (AUC) adjusted for age, BMI and HbA_{1c}. Effect sizes are standardized betas.

South Asians

pGDM vs CTR

Crude model

	TGD		
Predictors	*Estimates*	*CI*	*p*
(Intercept)	0.99	0.43 – 1.55	**0.001**
GDM [GDM]	-1.41	-2.08 – -0.74	**<0.001**
Observations	27		
R^2 / R^2 adjusted	0.430 / 0.408		

Model adjusted for BMI, age and HbA_{1c}

	TGD		
Predictors	*Estimates*	*CI*	*p*
(Intercept)	10959	-2.56 – 5.16	0.489
GDM [GDM]	-1.13	-1.99 – -0.28	**0.012**
BMI	-0.02	-0.07 – 0.02	0.307
Age	0.05	-0.04 – 0.13	0.272
HbA_{1c}	-0.04	-0.11 – 0.04	0.312
Observations	25		
R^2 / R^2 adjusted	0.529 / 0.435		

Nordics

pGDM vs CTR

Crude model

	TGD		
Predictors	*Estimates*	*CI*	*p*
(Intercept)	0.97	0.44 – 1.51	**0.001**
GDM [GDM]	-1.46	-2.11 – -0.80	**<0.001**
Observations	24		
R^2 / R^2 adjusted	0.493 / 0.470		

Model adjusted for BMI, age and HbA_{1c}

	TGD		
Predictors	*Estimates*	*CI*	*p*
(Intercept)	27395	-2.24 – 5.73	0.370
GDM [GDM]	-1.06	-1.95 – -0.18	**0.021**
BMI	-0.09	-0.19 – 0.00	0.056
Age	0.00	-0.06 – 0.07	0.887
HbA_{1c}	0.04	-0.06 – 0.13	0.467
Observations	24		
R^2 / R^2 adjusted	0.600 / 0.515		

ESM Table 2.

Sensitivity analyses for EGP (AUC) adjusted for age, BMI and HbA_{1c}. Effect sizes are standardized betas.

South Asians

pGDM vs CTR

Crude model

		EGP	
Predictors	*Estimates*	*CI*	*p*
(Intercept)	-0.38	-1.10 – 0.34	0.289
GDM [GDM]	0.54	-0.32 – 1.39	0.209
Observations	27		
R^2 / R^2 adjusted	0.062 / 0.025		

Model adjusted for BMI, age and HbA_{1c}

		EGP	
Predictors	*Estimates*	*CI*	*p*
(Intercept)	-1.06	-6.54 – 4.41	0.690
GDM [GDM]	0.26	-0.95 – 1.47	0.660
BMI	0.05	-0.01 – 0.11	0.127
Age	-0.00	-0.12 – 0.12	0.938
HbA_{1c}	-0.01	-0.11 – 0.10	0.870
Observations	25		
R^2 / R^2 adjusted	0.166 / -0.001		

Nordics

pGDM vs CTR

Crude model

		EGP	
Predictors	*Estimates*	*CI*	*p*
(Intercept)	-0.94	-1.49 – -0.38	**0.002**
GDM [GDM]	1.41	0.73 – 2.08	**<0.001**
Observations	24		
R2 / R2 adjusted	0.458 / 0.433		

Model adjusted for BMI, age and HbA_{1c}

		EGP	
Predictors	*Estimates*	*CI*	*p*
(Intercept)	-2.88	-7.19 – 1.43	0.178
GDM [GDM]	1.2	0.24 – 2.16	**0.017**
BMI	0.01	-0.09 – 0.11	0.847
Age	-0.03	-0.09 – 0.04	0.418
HbA_{1c}	0.08	-0.03 – 0.18	0.151
Observations	24		
R^2 / R^2 adjusted	0.531 / 0.433		

South Asians vs Nordics

pGDM

Crude model

		EGP	
Predictors	*Estimates*	*CI*	*p*
(Intercept)	0.51	0.06 – 0.97	**0.028**
Etn [SA]	-0.94	-1.56 – -0.33	**0.004**
Observations	35		
R^2 / R^2 adjusted	0.228 / 0.204		

Model adjusted for BMI, age and HbA1c

		EGP	
Predictors	*Estimates*	*CI*	*p*
(Intercept)	-3.06	-6.63 – 0.51	0.090
Etn [SA]	-1.32	-1.96 – -0.67	**<0.001**
BMI	0.03	-0.01 – 0.08	0.151
Age	-0.01	-0.07 – 0.04	0.623
HbA1c	0.08	0.01 – 0.16	**0.027**
Observations	33		
R^2 / R^2 adjusted	0.430 / 0.349		

ESM Table 3.

Sensitivity analyses for GLSUP (%) adjusted for age, BMI and HbA_{1c}. Effect sizes are standardized betas.

South Asians

pGDM vs CTR

Crude model

	GLSUP		
Predictors	*Estimates*	*CI*	*p*
(Intercept)	-0.36	-1.08 – 0.36	0.311
GDM [GDM]	0.51	-0.34 – 1.37	0.229
Observations	27		
R^2 / R^2 adjusted	0.057 / 0.020		

Model adjusted for BMI, age and HbA $_{1c}$

	GLSUP		
Predictors	*Estimates*	*CI*	*p*
(Intercept)	-5.62	-9.57 – -1.66	**0.008**
GDM [GDM]	-0.59	-1.47 – 0.28	0.173
BMI	0.05	0.01 – 0.10	**0.029**
Age	0.02	-0.06 – 0.11	0.588
HbA_{1c}	0.09	0.02 – 0.17	**0.015**
Observations	25		
R^2 / R^2 adjusted	0.494 / 0.392		

Nordics

pGDM vs CTR

Crude model

	GLSUP		
Predictors	*Estimates*	*CI*	*p*
(Intercept)	-0.62	-1.29 – 0.05	0.069
GDM [GDM]	0.93	0.11 – 1.75	**0.029**
Observations	24		
R^2 / R^2 adjusted	0.200 / 0.163		

Model adjusted for BMI, age and HbA $_{1c}$

	GLSUP		
Predictors	*Estimates*	*CI*	*p*
(Intercept)	-4.43	-9.33 – 0.48	0.074
GDM [GDM]	0.08	-1.01 – 1.17	0.873
BMI	0.13	0.02 – 0.25	**0.027**
Age	0.04	-0.04 – 0.11	0.308
HbA_{1c}	-0.02	-0.14 – 0.11	0.784
Observations	24		
R^2 / R^2 adjusted	0.393 / 0.265		

South Asian controls vs Nordic pGDM

Crude model

	GLSUP		
Predictors	*Estimates*	*CI*	*p*
(Intercept)	-0.00	-0.53 – 0.53	1.000
Etn [SA]	0.00	-0.92 – 0.92	1.000
Observations	24		
R^2 / R^2 adjusted	0.000 / -0.045		

Model adjusted for BMI, age and HbA $_{1c}$

	GLSUP		
Predictors	*Estimates*	*CI*	*p*
(Intercept)	-8.24	-12.43 – -4.05	**0.001**
Etn [SA]	1.09	0.20 – 1.97	**0.019**
BMI	0.14	0.06 – 0.23	**0.002**
Age	0.04	-0.03 – 0.10	0.239
HbA_{1c}	0.07	-0.02 – 0.17	0.117
Observations	24		
R^2 / R^2 adjusted	0.520 / 0.420		

South Asian pGDM vs Nordic controls

Crude model

	GLSUP		
Predictors	*Estimates*	*CI*	*p*
(Intercept)	-0.94	-1.52 – -0.37	**0.003**
Etn [SA]	1.34	0.65 – 2.03	**<0.001**
Observations	27		
R^2 / R^2 adjusted	0.390 / 0.366		

Model adjusted for BMI, age and HbA $_{1c}$

	GLSUP		
Predictors	*Estimates*	*CI*	*p*
(Intercept)	-2.60	-6.87 – 1.68	0.220
Etn [SA]	0.68	-0.16 – 1.53	0.106
BMI	0.04	-0.01 – 0.08	0.089
Age	-0.02	-0.11 – 0.06	0.587
HbA_{1c}	0.05	-0.03 – 0.12	0.229
Observations	25		
R^2 / R^2 adjusted	0.552 / 0.463		

ESM Table 4. Regression analyses of TGD, EGP and GLSUP as a function of BMI by ethnicity, adjusted for pGDM status. Estimates are standardized betas.

TGD

Predictors	*Estimates*	*CI*	*p*
(Intercept)	3.39	1.42 – 5.37	**0.001**
BMI	-0.10	-0.17 – -0.02	**0.016**
Etn [SA]	-2.04	-4.28 – 0.21	0.074
GDM [GDM]	-1.03	-1.55 – -0.51	**<0.001**
BMI × Etn [SA]	0.07	-0.01 – 0.15	0.082
Observations	51		
R^2 / R^2 adjusted	0.512 / 0.469		

EGP

Predictors	*Estimates*	*CI*	*p*
(Intercept)	-2.13	-4.72 – 0.46	0.104
BMI	0.09	-0.02 – 0.19	0.094
Etn [SA]	2.53	-0.41 – 5.48	0.090
GDM [GDM]	0.11	-0.58 – 0.79	0.756
BMI × Etn [SA]	-0.11	-0.22 – -0.00	**0.041**
Observations	51		
R^2 / R^2 adjusted	0.160 / 0.087		

GLSUP

Predictors	*Estimates*	*CI*	*p*
(Intercept)	-3.59	-5.64 – -1.53	**0.001**
BMI	0.11	0.03 – 0.19	**0.008**
Etn [SA]	1.89	-0.45 – 4.22	0.111
GDM [GDM]	0.38	-0.17 – 0.92	0.169
BMI × Etn [SA]	-0.05	-0.13 – 0.03	0.237
Observations	51		
R^2 / R^2 adjusted	0.470 / 0.424		

ESM Table 5. Correlations in South Asians.

	TGD		EGP		GLSUP%	
	r	*p*	r	*p*	r	*p*
Insulin	-0.3765205	0.0528921	-0.0683159	0.7349272	0.5539928	0.0027161
WHtR	-0.3053931	0.1213686	-0.1864035	0.3518825	0.6373541	0.0003495
WHR	-0.4822798	0.010846	-0.0271623	0.8930188	0.3811299	0.0498263
Fat (%)	-0.2708507	0.1717856	-0.2588434	0.1923366	0.6003638	0.0009303
Fat (kg)	-0.2435944	0.2207987	-0.2294415	0.2496341	0.6527207	0.000224
FFM (kg)	-0.2116471	0.2892463	-0.2696341	0.1737946	0.6431558	0.0002963
Truncal fat (%)	-0.2386963	0.2305123	-0.3064581	0.1200099	0.5338356	0.0041322
Truncal fat (kg)	-0.2766224	0.1624771	-0.2852906	0.1491771	0.6671081	0.0001444
Truncal FFM (kg)	-0.2270201	0.2548039	-0.2550228	0.199217	0.6326884	0.0003982
Systolic BP	-0.1667294	0.4058618	0.085583	0.6712449	0.3457622	0.0773008
Diastolic BP	-0.5788699	0.001559	0.2713369	0.1709874	0.5235076	0.0050738
HR	-0.0344756	0.8644485	-0.0205338	0.9190279	0.1819658	0.3636682
HbA_{1c}	-0.2353144	0.257497	-0.0276656	0.8955609	0.5046722	0.0100878
Fasting glucose	-0.2765723	0.1713922	0.1382137	0.500733	0.3253705	0.1048118
Total-C	-0.0553032	0.7928895	-0.013878	0.9475049	0.1335388	0.5245349
HDL-C	0.4890539	0.0131041	0.3079181	0.1342822	-0.0306027	0.8845423
LDL-C	-0.2228983	0.2841783	-0.1476105	0.4813462	0.151482	0.46979
TG	-0.3839058	0.0581468	-0.1621113	0.4388119	0.0587991	0.7801025
ASAT	0.0916581	0.6560883	-0.1014939	0.6217693	-0.1210405	0.555857
ALAT	0.1936114	0.3432903	-0.286359	0.1561204	-0.0829737	0.6869682
Creatinine	0.0627108	0.7608712	-0.4476297	0.0218451	0.2408036	0.2360131
eGFR	-0.0835728	0.6848214	0.4997826	0.0093293	-0.3136553	0.1186767

ESM Table 6. Correlations in Nordics.

	TGD		EGP		GLSUP%	
	r	*p*	r	*p*	r	*p*
Insulin	-0.8345293	3.99E-07	0.410524	0.0463032	0.5296804	0.0077687
WHtR	-0.833913	4.15E-07	0.4591304	0.0240151	0.8069565	1.90E-06
WHR	-0.193913	0.3639189	0.1173913	0.5848655	0.3930435	0.0574331
Fat (%)	-0.803653	2.25E-06	0.5018482	0.012465	0.5466406	0.0057105
Fat (kg)	-0.8173913	1.09E-06	0.5095652	0.0109754	0.5321739	0.0074322
FFM (kg)	-0.4359366	0.0332152	0.2745269	0.1942124	0.3302154	0.1150403
Truncal fat (%)	-0.7791304	7.26E-06	0.5113043	0.0106609	0.4930435	0.0143637
Truncal fat (kg)	-0.7984345	2.93E-06	0.5179387	0.0095285	0.4983692	0.0131889
Truncal FFM (kg)	-0.3690167	0.0759745	0.2280245	0.2838769	0.342907	0.1009242
Systolic BP	-0.6363637	0.0008289	0.2844715	0.1778981	0.3484124	0.0952171
Diastolic BP	-0.5547097	0.0049049	0.3484882	0.0951402	0.2693061	0.2031735
HR	-0.4884733	0.015439	0.1909526	0.3714299	0.0665507	0.7573456
HbA_{1c}	-0.3062175	0.1455869	0.480324	0.0175193	0.3014055	0.1523472
Fasting glucose	-0.437229	0.0326381	0.6068024	0.0016671	0.3679176	0.0769198
Total-C	-0.2540866	0.2308778	-0.1019833	0.6353698	-0.060144	0.7801162
HDL-C	0.6956408	0.0001606	-0.3188719	0.1288333	-0.5399331	0.0064618
LDL-C	-0.5623385	0.0042333	0.1599831	0.4552179	0.2641683	0.2122614
TG	-0.8629637	5.77E-08	0.2583645	0.222851	0.521975	0.0088897
ASAT	0.1695176	0.4393669	0.0232963	0.9159724	-0.1769526	0.4192556
ALAT	-0.0888226	0.6798057	0.2463406	0.2458926	0.0831345	0.6993471
Creatinine	0.329553	0.1158148	-0.2793561	0.1861671	-0.6337893	0.0008834
eGFR	-0.1683392	0.431691	0.1696475	0.4280677	0.447887	0.0281775

ESM Table 7. Correlations in South Asians with pGDM.

	TGD		EGP		GLSUP%	
	r	*p*	r	*p*	r	*p*
Insulin	-0.3313826	0.1657674	0.2202552	0.3648878	0.5413808	0.0166704
WHtR	-0.2708035	0.2621247	0.0513032	0.8347759	0.5771604	0.0096704
WHR	-0.5079084	0.0264095	0.3731901	0.1155472	0.2522882	0.297403
Fat (%)	-0.2964615	0.2177786	-0.0726521	0.7675552	0.5337851	0.018578
Fat (kg)	-0.213718	0.3796477	-0.0771587	0.7535497	0.6104157	0.0055082
FFM (kg)	-0.1790937	0.463182	-0.147257	0.5474429	0.6388102	0.0032391
Truncal fat (%)	-0.2700274	0.2635483	-0.1543739	0.5280284	0.4155592	0.0768205
Truncal fat (kg)	-0.2782776	0.2486631	-0.1285226	0.6000228	0.6074387	0.0058071
Truncal FFM (kg)	-0.1916475	0.4318753	-0.1355927	0.5799368	0.6255332	0.0041779
Systolic BP	-0.0855407	0.7276983	0.2646604	0.2735246	0.2759563	0.2527961
Diastolic BP	-0.656251	0.002277	0.4465173	0.055308	0.7048006	0.0007526
HR	-0.0470497	0.8483165	0.0037295	0.9879103	0.2327081	0.3376906
HbA_{1c}	-0.1228131	0.638651	0.4885997	0.0465784	0.4765707	0.0531047
Fasting glucose	-0.1937885	0.4410045	0.4914394	0.038336	0.3079887	0.2137297
Total-C	0.0371971	0.8872903	0.0808423	0.7577496	0.2022063	0.4363888
HDL-C	0.5585424	0.0197836	-0.0722706	0.7828227	0.1061125	0.6852309
LDL-C	-0.173283	0.5059842	0.1253232	0.6317541	0.1414715	0.588088
TG	-0.3380287	0.1844971	-0.0007628	0.9976817	0.022811	0.930752
ASAT	0.139969	0.5796165	-0.3344838	0.174883	0.0203028	0.9362682
ALAT	0.2951886	0.2343615	-0.3398639	0.1676198	-0.0129993	0.9591711
Creatinine	-0.0203462	0.9361321	-0.3327057	0.1773292	0.2118225	0.3987778
eGFR	-0.0555825	0.8266066	0.3392709	0.1684102	-0.1860472	0.4598126

ESM Table 8. Correlations in Nordics with pGDM.

	TGD		EGP		GLSUP%	
	r	p	r	p	r	p
Insulin	-0.7970588	0.0002181	0.0382353	0.8881977	0.4617647	0.0717684
WHtR	-0.6441176	0.0070815	0.3235294	0.2215741	0.7941176	0.0002392
WHR	-0.2823529	0.2893498	0.2205882	0.4116664	0.5617647	0.023537
Fat (%)	-0.5033114	0.0468747	0.0603385	0.8243317	0.1574688	0.5602741
Fat (kg)	-0.6941176	0.0028537	0.0852941	0.7534651	0.3205882	0.2260454
FFM (kg)	-0.612215	0.0117093	0.1766005	0.5129356	0.5783666	0.0189273
Truncal fat (%)	-0.4647059	0.0697437	0.0176471	0.9482802	0.0323529	0.9053195
Truncal fat (kg)	-0.6519501	0.0062063	0.0515085	0.8497437	0.2266373	0.3986266
Truncal FFM (kg)	-0.6004417	0.0139183	0.1751288	0.5165099	0.6239884	0.0097857
Systolic BP	-0.5493379	0.0275183	-0.0810016	0.7655401	0.2209133	0.4109599
Diastolic BP	-0.5783666	0.0189273	-0.111847	0.68005	0.0662252	0.8074793
HR	-0.5518765	0.0266659	-0.236939	0.3769367	-0.1913172	0.477834
HbA_{1c}	0.0922684	0.7339609	0.1205443	0.6565426	0.2098363	0.4353886
Fasting glucose	-0.0619472	0.8197189	0.5840733	0.0175162	0.1209445	0.6554679
Total-C	-0.2998552	0.2591776	-0.2215679	0.4095395	0.0812416	0.7648636
HDL-C	0.4618568	0.0717043	0.1619498	0.5490203	-0.2879108	0.2795504
LDL-C	-0.3574629	0.1740549	-0.2023654	0.4522743	0.2038425	0.4489099
TG	-0.9180874	5.33E-07	-0.0782293	0.7733657	0.4841361	0.0573986
ASAT	0.0626681	0.8244128	0.1754706	0.5316293	0.003581	0.9898943
ALAT	-0.1186991	0.6615056	0.3026826	0.2544927	0.2522355	0.3459564
Creatinine	0.0639445	0.8139994	-0.0520478	0.8481872	-0.4297665	0.0966306
eGFR	-0.0752773	0.7817214	0.0797053	0.7691966	0.2833968	0.2874939

ESM Table 9. Correlations in South Asian controls.

	TGD		EGP		GLSUP%	
	r	*p*	r	*p*	r	*p*
Insulin	-0.3540828	0.3894989	-0.4314439	0.285825	0.4750088	0.2342622
WHtR	0.0361805	0.9322208	-0.0124779	0.9766064	0.7328669	0.0386187
WHR	0.201866	0.6316581	-0.167162	0.6923611	0.4808962	0.2276907
Fat (%)	0.0911019	0.8301266	-0.1254778	0.767187	0.5845324	0.1280639
Fat (kg)	0.0814733	0.8479122	-0.1924752	0.6479231	0.6883031	0.0591123
FFM (kg)	-0.025557	0.9521016	-0.510413	0.1962016	0.7202246	0.043903
Truncal fat (%)	0.10731	0.8003331	-0.1846955	0.6614909	0.5730157	0.1376138
Truncal fat (kg)	0.0965639	0.8200651	-0.2126898	0.6130703	0.68012	0.0634525
Truncal FFM (kg)	-0.1194182	0.7782105	-0.4834326	0.2248891	0.7215406	0.0433337
Systolic BP	-0.2829558	0.497096	0.1764807	0.6759053	0.290344	0.4854262
Diastolic BP	-0.2861044	0.4921094	0.0664345	0.8758013	0.1246968	0.7686059
HR	0.0622993	0.8834907	-0.051395	0.9038039	0.0622238	0.8836312
HbA_{1c}	-0.4958611	0.2114206	-0.2773727	0.5059853	0.4420579	0.2727922
Fasting glucose	-0.4629951	0.2479679	0.128684	0.761368	0.0870653	0.8375758
Total-C	-0.4901543	0.2175514	-0.0254168	0.9523641	-0.1001617	0.813449
HDL-C	-0.1298034	0.7593386	0.7731021	0.0244592	-0.0133518	0.9749684
LDL-C	-0.2575402	0.5380396	-0.3364162	0.4151964	-0.0282885	0.9469873
TG	-0.498733	0.2083698	-0.2192694	0.6018576	-0.1191467	0.7787052
ASAT	-0.2056105	0.6252079	0.2392551	0.5682222	-0.3840129	0.3476303
ALAT	0.0928391	0.8269244	0.0201817	0.9621696	-0.5231418	0.1833806
Creatinine	0.351418	0.3933292	-0.8404711	0.0089742	0.4180822	0.302653
eGFR	-0.2667107	0.5231267	0.784637	0.0211122	-0.5205591	0.1859447

ESM Table 10. Correlations in Nordic controls.

	TGD		EGP		GLSUP%	
	r	*p*	r	*p*	r	*p*
Insulin	-0.6428571	0.0855589	0.7380952	0.0365528	0.1666667	0.6932388
WHtR	-0.4047619	0.3198886	-0.3809524	0.3518126	-0.0714286	0.8665263
WHR	0.2142857	0.6103444	-0.3571429	0.3851206	0.3333333	0.4197531
Fat (%)	-0.8095238	0.0149027	0.1190476	0.7788857	0.1904762	0.6514015
Fat (kg)	-0.6428571	0.0855589	-0.0714286	0.8665263	-0.0238095	0.955374
FFM (kg)	0.2619048	0.5309229	0	1	-0.3095238	0.4556449
Truncal fat (%)	-0.7857143	0.0208151	0.2142857	0.6103444	0.1666667	0.6932388
Truncal fat (kg)	-0.547619	0.1600256	0.2380952	0.5701563	0	1
Truncal FFM (kg)	0.2619048	0.5309229	0	1	-0.3095238	0.4556449
Systolic BP	-0.5952381	0.1195298	0.547619	0.1600256	-0.0952381	0.8225054
Diastolic BP	-0.1666667	0.6932388	0.9047619	0.0020083	0.0952381	0.8225054
HR	-0.6666667	0.0709877	0.452381	0.2604048	0.547619	0.1600256
HbA_{1c}	-0.0602453	0.887313	0.0240981	0.9548335	-0.4940118	0.2133973
Fasting glucose	0.349423	0.3962077	-0.8434347	0.0085032	0.0481963	0.9097718
Total-C	-0.243975	0.5603756	-0.29277	0.4816178	-0.8051176	0.0159046
HDL-C	0.0240981	0.9548335	-0.5060608	0.2006907	0.0361472	0.932283
LDL-C	-0.5123475	0.1942234	0.0731925	0.8632534	-0.7319251	0.0389983
TG	-0.3928229	0.33572	0.1964114	0.6410902	-0.2332386	0.5782791
ASAT	0.5	0.2070313	-0.1904762	0.6514015	-0.7380952	0.0365528
ALAT	0.503003	0.2038767	-0.2395253	0.5677722	-0.6706707	0.0686927
Creatinine	0.1686869	0.6896608	-0.5663062	0.1433541	-0.8193366	0.0128165
eGFR	-0.2619048	0.5309229	0.5952381	0.1195298	0.7857143	0.0208151

ESM Table 11. Interaction effects between South Asians and Nordics with pGDM.

	TGD		EGP		GLSUP%	
	beta	*p*	beta	*p*	beta	*p*
Insulin	-0.236	0.487	-0.014	0.452	0.080	0.393
WHtR	-45.435	0.842	-21.397	0.085	-12.257	0.828
WHR	-523.612	0.030	0.662	0.962	-0.037	1.000
Fat (%)	-0.959	0.793	-0.214	0.306	0.977	0.343
Fat (kg)	0.726	0.762	-0.167	0.211	0.193	0.755
FFM (kg)	0.687	0.867	-0.203	0.373	0.007	0.995
Truncal fat (%)	-0.884	0.797	-0.165	0.399	0.956	0.348
Truncal fat (kg)	-0.533	0.911	-0.256	0.344	1.488	0.245
Truncal FFM (kg)	0.136	0.985	-0.367	0.374	0.236	0.898
Systolic BP	0.090	0.962	0.038	0.714	0.403	0.475
Diastolic BP	-4.487	0.014	0.136	0.272	1.551	0.007
HR	0.133	0.950	0.098	0.390	0.897	0.156
HbA_{1c}	-2.515	0.623	-0.094	0.722	0.831	0.481
Fasting glucose	-17.363	0.592	-3.036	0.053	1.120	0.889
Total-C	7.739	0.805	1.218	0.471	2.222	0.772
HDL-C	83.278	0.144	-0.779	0.833	20.309	0.225
LDL-C	-17.552	0.597	1.309	0.473	-1.863	0.820
TG	-20.856	0.465	0.582	0.726	-6.086	0.416
ASAT	1.630	0.557	-0.349	0.010	-0.377	0.588
ALAT	2.010	0.225	-0.223	0.009	-0.349	0.412
Creatinine	-0.870	0.754	-0.015	0.919	1.163	0.091
eGFR	0.108	0.957	-0.004	0.970	-0.652	0.192

ESM Table 12. Interaction effects between South Asian and Nordic control.

	TGD		EGP		GLSUP%	
	beta	*p*	beta	*p*	beta	*p*
Insulin	1.004	0.311	-0.336	0.028	0.288	0.590
WHtR	219.368	0.670	165.667	0.014	208.516	0.296
WHR	122.958	0.782	39.123	0.515	312.589	0.162
Fat (%)	3.912	0.109	-0.194	0.624	1.282	0.281
Fat (kg)	2.401	0.267	-0.209	0.536	1.556	0.102
FFM (kg)	-0.409	0.922	-0.479	0.442	4.574	0.013
Truncal fat (%)	3.002	0.127	-0.249	0.427	1.092	0.257
Truncal fat (kg)	4.083	0.261	-0.465	0.410	2.665	0.099
Truncal FFM (kg)	-2.943	0.714	-0.787	0.512	9.008	0.011
Systolic BP	0.802	0.576	-0.222	0.319	0.528	0.526
Diastolic BP	-0.304	0.868	-0.318	0.201	0.213	0.835
HR	1.409	0.391	-0.179	0.481	0.039	0.968
HbA_{1c}	-3.223	0.530	-0.364	0.662	2.916	0.288
Fasting glucose	-46.844	0.240	9.312	0.110	5.158	0.819
Total-C	-17.605	0.560	0.534	0.917	0.164	0.993
HDL-C	-21.842	0.707	16.639	0.039	-4.668	0.882
LDL-C	-2.124	0.944	-2.648	0.581	4.182	0.805
TG	21.674	0.671	-11.026	0.201	-1.784	0.955
ASAT	-4.475	0.134	0.768	0.078	-0.721	0.646
ALAT	-1.697	0.382	0.392	0.174	-0.741	0.436
Creatinine	0.072	0.974	0.221	0.424	1.479	0.203
eGFR	0.303	0.841	-0.195	0.277	-1.230	0.109

ESM Figure 1

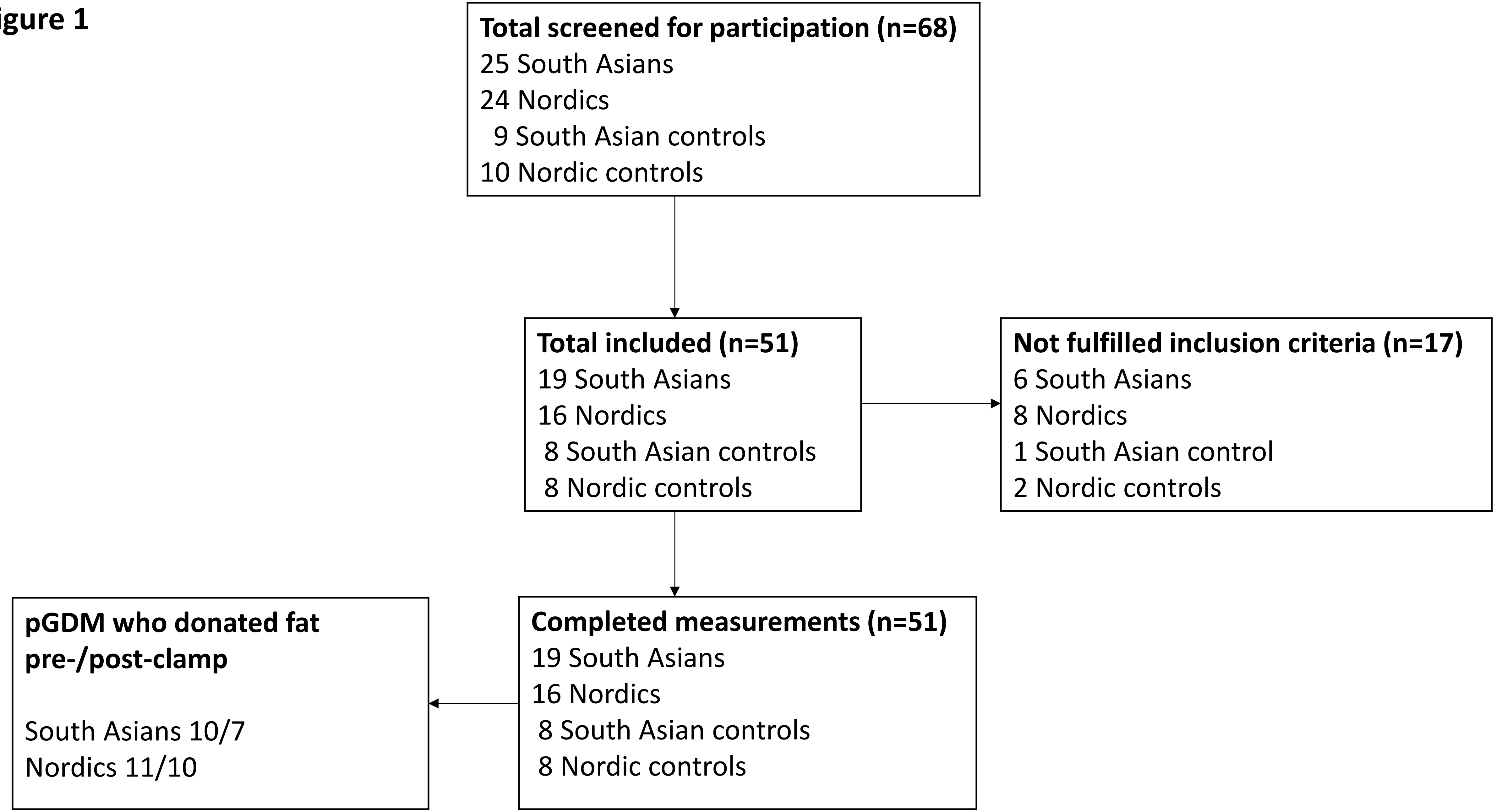
Total screened for participation (n=68)
25 South Asians
24 Nordics
9 South Asian controls
10 Nordic controls
Total included (n=51)
19 South Asians
16 Nordics
8 South Asian controls
8 Nordic controls
Not fulfilled inclusion criteria (n=17)
6 South Asians
8 Nordics
1 South Asian control
2 Nordic controls
Completed measurements (n=51)
19 South Asians
16 Nordics
8 South Asian controls
8 Nordic controls
pGDM who donated fat
pre-/post-clamp
South Asians 10/7
Nordics 11/10
